# The *Pseudomonas aeruginosa* antimetabolite L -2-amino-4-methoxy-*trans*-3-butenoic acid (AMB) is made from glutamate and two alanine residues via a thiotemplate-linked tripeptide precursor

**DOI:** 10.3389/fmicb.2015.00170

**Published:** 2015-03-12

**Authors:** Nelson Rojas Murcia, Xiaoyun Lee, Patrice Waridel, Alessandro Maspoli, Heidi J. Imker, Tiancong Chai, Christopher T. Walsh, Cornelia Reimmann

**Affiliations:** ^1^Department of Fundamental Microbiology, University of Lausanne, LausanneSwitzerland; ^2^Protein Analysis Facility, University of Lausanne, LausanneSwitzerland; ^3^Department of Biological Chemistry and Molecular Pharmacology, Harvard Medical School, Boston, MAUSA

**Keywords:** *Pseudomonas*, toxin, oxyvinylglycine, secondary metabolite, thiotemplate

## Abstract

The *Pseudomonas aeruginosa* toxin L-2-amino-4-methoxy-*trans*-3-butenoic acid (AMB) is a non-proteinogenic amino acid which is toxic for prokaryotes and eukaryotes. Production of AMB requires a five-gene cluster encoding a putative LysE-type transporter (AmbA), two non-ribosomal peptide synthetases (AmbB and AmbE), and two iron(II)/α-ketoglutarate-dependent oxygenases (AmbC and AmbD). Bioinformatics analysis predicts one thiolation (T) domain for AmbB and two T domains (T1 and T2) for AmbE, suggesting that AMB is generated by a processing step from a precursor tripeptide assembled on a thiotemplate. Using a combination of ATP-PP_i_ exchange assays, aminoacylation assays, and mass spectrometry-based analysis of enzyme-bound substrates and pathway intermediates, the AmbB substrate was identified to be L-alanine (L-Ala), while the T1 and T2 domains of AmbE were loaded with L-glutamate (L-Glu) and L-Ala, respectively. Loading of L-Ala at T2 of AmbE occurred only in the presence of AmbB, indicative of a *trans* loading mechanism. *In vitro* assays performed with AmbB and AmbE revealed the dipeptide L-Glu-L-Ala at T1 and the tripeptide L-Ala-L-Glu-L-Ala attached at T2. When AmbC and AmbD were included in the assay, these peptides were no longer detected. Instead, an L-Ala-AMB-L-Ala tripeptide was found at T2. These data are in agreement with a biosynthetic model in which L-Glu is converted into AMB by the action of AmbC, AmbD, and tailoring domains of AmbE. The importance of the flanking L-Ala residues in the precursor tripeptide is discussed.

## INTRODUCTION

*Pseudomonas aeruginosa* is an opportunistic pathogen often affecting patients who suffer from compromised antimicrobial barriers, as for instance the genetic disease cystic fibrosis (Lyczak et al., 2002). One of the many toxins that this bacterium produces is L-2-amino-4-methoxy-*trans*-3-butenoic acid (AMB), a non-proteinogenic amino acid belonging to a small group of natural compounds known as oxyvinylglycines (Berkowitz et al., 2006). Other members of this group are aminoethoxyvinylglycine (AVG), dimethyliminooxyvinylglycine, and guanidinooxyvinylglycine from *Streptomyces* spp. (Pruess et al., 1974; Hirata et al., 1993), rhizobitoxin made by *Bradyrhizobium japonicum* and *P. andropogonis* (Owens et al., 1972; Mitchell et al., 1986), as well as the recently identified 4-formylaminooxyvinylglycine (FVG) isolated from *P. fluorescens* WH6 (McPhail et al., 2010). Oxyvinylglycines irreversibly inhibit pyridoxal phosphate (PLP)-dependent enzymes and thus have multiple targets in bacteria, animals, and plants (Berkowitz et al., 2006). A prominent plant target is the ethylene biosynthesis enzyme ACC synthase, which is inhibited by AVG. Commercially available under the name of Retain®;, AVG is widely used for the regulation of fruit set in orchard crops. Another example with potential for an agricultural application is FVG. This oxyvinylglycine is a natural herbicide which blocks the germination of a large variety of grassy weed species (Banowetz et al., 2008).

Pyridoxal phosphate-dependent enzymes are also targeted by AMB. Isolated originally as a growth inhibitor of *Bacillus subtilis* (Scannell et al., 1972) and *Escherichia coli* (Sahm et al., 1973), AMB was shown to inhibit apartate aminotransferase in pigs (Rando, 1974; Rando et al., 1976) and rat hepatocytes (Smith and Freeland, 1981; Cornell et al., 1984), tryptophane synthase in *E. coli* (Miles, 1975), δ-aminolevulinic acid synthetase in rats (Dashman and Kamm, 1979), and serine hydroxylmethyl transferase in Walker carcinoma (Tisdale, 1981). Reversible inhibition of L-methionine tRNA aminoacylation was also reported, suggesting that AMB can function as a methionine antimetabolite (Matoo et al., 1979). We recently evaluated the importance of AMB as a *P. aeruginosa* virulence factor using an *Acanthamoeba castellanii* cell model (Lee et al., 2012). Although AMB was found to inhibit growth and to induce cyst formation, the effective concentrations were rather high, making a strong contribution of AMB to the virulence of *P. aeruginosa* unlikely. AMB may be more important during interactions of *P. aeruginosa* with other microbes and it is interesting to note in this respect that AMB can inhibit the growth of important plant and animal pathogens such as *Erwinia amylovora* (Lee et al., 2013a) and *Staphylococcus aureus* (our unpublished observation).

**FIGURE 1:**
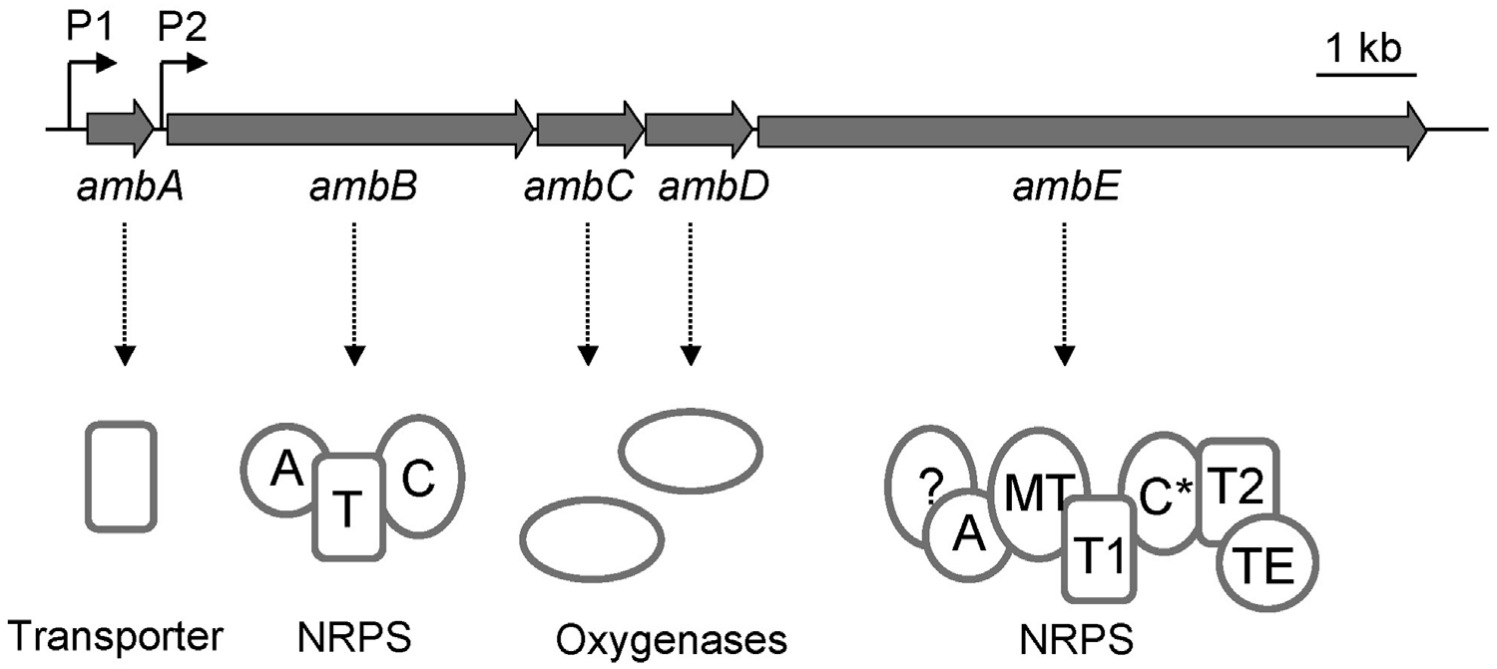
**Organization of the *amb* gene cluster in the *Pseudomonas aeruginosa* strain PAO1.** The *ambA* gene encodes a putative LysE-type transmembrane protein potentially involved in AMB export, *ambB* and *ambE* code for non-ribosomal peptide synthetases (NRPS), and *ambC* and *ambD* specify two putative iron (II)/α-ketoglutarate-dependent oxygenases. The modular structures of AmbB and AmbE are shown with domains for adenylation (A), thiolation (T), condensation (C), methylation (MT), and thioester cleavage (TE). Note that AmbE may have an additional domain of unknown function at its N-terminus and that the C domain (indicated as C*) is atypical (see text for details).

Transposon mutagenesis and reverse genetics have previously led to the identification of the *P. aeruginosa ambABCDE* gene cluster which comprises two transcriptional units (Lee et al., 2010, 2013a; **Figure 1**). Transfer of this cluster to strains devoid of *amb* genes, such as *P. aeruginosa* PA7, or *P. fluorescens* CHA0, rendered them capable of synthesizing AMB (Lee et al., 2010, 2013a), demonstrating that these genes are both essential and sufficient for AMB production. Bioinformatics analyses indicate that the first transcriptional unit, *ambA*, encodes a putative LysE-type transporter and may thus account for AMB secretion. The second transcriptional unit specifies four proteins predicted to be involved in AMB biosynthesis. Two of these, AmbB and AmbE, present the typical modular structure of non-ribosomal peptide synthetases (NRPSs; **Figure 1**). The gene products of *ambC* and *ambD* are predicted to belong to the family of iron(II)/α-ketoglutarate-dependent oxygenases. Most members of this family catalyze hydroxylation of a substrate coupled to the oxidative decarboxylation of an α-ketoglutarate cofactor using iron (II) as the redox catalyst. However, other members also catalyze reactions such as oxidative desaturation or cyclization (Hausinger, 2004).

Based on the observation that the Amb assembly line contains three thiolation domains, (i.e., attachment points for amino acid precursors, see **Figure 1**), we postulate that AMB biosynthesis proceeds via a precursor tripeptide. Using *in vitro* methods and analysis of enzyme-attached substrates and pathway intermediates by mass spectrometry (MS), we identified the building blocks of AMB biosynthesis and we present a model of how the AMB precursor tripeptide may be assembled.

## MATERIALS AND METHODS

### BACTERIAL STRAINS, PLASMIDS, AND CULTURE CONDITIONS

Strains and plasmids used in this study are listed in **Table 1**. Bacteria were routinely cultivated at 37^∘^C on nutrient agar and in nutrient yeast broth (Stanisich and Holloway, 1972). To facilite uptake of heterologous DNA during conjugation and transformation, *P. aeruginosa* was grown at 43^∘^C. When necessary, antibiotics were added to the media of *E. coli* at the following concentrations: ampicillin at 100 μg ml^-1^, chlorampheniol (Cm) at 30 μg ml^-1^, kanamycin (Km), and tetracycline (Tc) at 25 μg ml^-1^ each. For selection of Tc-resistant plasmids in *P. aeruginosa*, Tc was used at 125 μg ml^-1^. Counterselection of *E. coli* donor cells during mutant construction occurred with Cm at 10 μg ml^-1^; mutant enrichment was performed with Tc at 20 μg ml^-1^ and carbenicillin (Cb) at 2 mg ml^-1^. For protein purification, cultures were induced with isopropyl β-D-1-thiogalactopyranoside (IPTG) as detailed below.

**Table 1:** Bacterial strains and plasmids.

Name	Relevant characteristics	Reference/Source
***Escherichia coli* strains**
BL21(DE3)	F^-^*, ompT, gal, dcm, lon, hsdS_B_*(r_B_^-^ m_B_^-^), λ(DE3)	Novagen
BL21(DE3)/pLys	F^-^*, ompT, gal, dcm, lon, hsdS_B_*(r_B_^-^ m_B_^-^), λ(DE3)/pLys; Cm^r^	Novagen
DH5α	*recA1 endA1 hsdR17 deoR thi-1 supE44 gyrA96 relA1* Δ(*lacZYA-argF*) *U169* (ϕ80d*lacZ*Δ*M15*)	Sambrook and Russell (2001)
K12	Wild type	Tatum and Lederberg (1947)
***Pseudomonas aeruginosa* strains**
PAO1	Wild type	ATCC 15692
PAO6932	*ambB*_T2302G_ (specifying AmbB_S768A_)	This study
PAO6934	*ambE*_T3856G_ (specifying AmbE_S1286A_)	This study
PAO6935	*ambE*_T5455G,G5457A_ (specifying AmbE_S1819A_)	This study
**Plasmids**
pET-27b	P*_ T7_* expression vector for C-terminal His_6_-tagged proteins in *E. coli*; Km^r^	Novagen
pET-28a	P*_T7_* expression vector for N-terminal His_6_-tagged proteins in *E. coli*; Km^r^	Novagen
pET-29Sfp	Expression plasmid for Sfp-His_6_; Km^r^	Yin et al. (2006)
pME497	Mobilizing plasmid, Ap^r^	Voisard et al. (1988)
pME3087	Suicide vector, ColE1 replicon; Tc^r^	Voisard et al. (1988)
pME9713	pET-28a-based expression plasmid for His_6_-AmbB; Km^r^	This study
pME9714	pET-27b-based expression plasmid for AmbE-His_6_; Km^r^	This study
pME9717	pET-27b-based expression plasmid for AmbD-His_6_; Km^r^	This study
pME9718	pET-27b-based expression plasmid for AmbC-His_6_; Km^r^	This study
pME10317	pET-27b-based expression vector for AmbE_S1819A_-His_6_; Km^r^	This study
pME10326	pME3087 derivative for generating a S768A mutation in the thiolation domain of AmbB (replacing codon GGT against GGC)	This study
pME10327	pET-28a-based expression plasmid for His_6_-AmbB_S768A_; Km^r^	This study
pME10328	pET-27b-based expression plasmid for AmbE_S1958A_-His_6_; Km^r^	This study
pME10330	pET-27b-based expression plasmid for AmbE_D644A,K1230T,S1958A_-His_6_; Km^r^	This study
pME10337	pET-27b-based expression plasmid for AmbE_S1286A_-His_6_; Km^r^	This study
pME10338	pME3087 derivative for generating a S1286A mutation in the first thiolation domain of AmbE (replacing codon TCC against GCC)	This study
pME10341	pME3087 derivative for generating a S1819A mutation in the second thiolation domain of AmbE (replacing codon TCG against GCA)	This study

### DNA MANIPULATIONS AND SEQUENCING

DNA manipulations were done according to standard procedures (Sambrook and Russell, 2001). Plasmid DNA was prepared using Jetstar (Genomed GmbH) and QIAprep Spin Miniprep (Qiagen, Inc.) kits; DNA fragments were gel-purified with the Invisorb®; fragment cleanUp kit from Invitek. Bacterial transformations were done by electroporation (Farinha and Kropinski, 1990). Constructs involving PCR techniques (for oligonucleotides see **Table 2**) were verified by commercial sequence analysis carried out at GATC Biotech.

**Table 2:** Oligonucleotides (5′→3′)^**a**^.

2302Amut-1	AAGCGCGACGGTGCCACC
2302Amut-2	AGCGCGCGACGGTCGAGC**G**TGCCTTCGGCGGTCAGC
2302Amut-3	GCTGACCGCCGAAGGCA**C**GCTCGACCGTCGCGCGCT
2302Amut-4	CCGGACTCGCCGGAGCGG
XL007	TCAGTCACATATGAGTGCGTCAGAAGACCTG (NdeI)
XL009	GTCAGCTAGCAGAGCGATGCAGGAGCGACA (NheI)
XL010	ACTGGAATTCCGGGGTCGTCAGGAAGCGTT (EcoRI)
XL032	ACTGAGATCTGGAAGAGCGTGCTGAAAC (BglII)
XL033	GTCAGCTAGCGGTTGCCAGGTTCGCCG (NheI)
XL034	ACTGACTCATATGAGCGCCTCGTTCAG (NdeI)
XL036	ACTGACTCATATGGAACGAACAGCTCCC (NdeI)
XL042	GTCAGAATTCTCAGTGGTGGTGGTGGTGGTGGGGTTGGTGGACAT (EcoRI)
XL044	GTCAGAATTCTCAATGATGATGATGATGATGTGCGGCACCTCC (EcoRI)
XL046	TCAGTCACATATG GTGGAATCCCTGGTGGCCGCGT (NdeI)
XL094	CAATTCGGCGATCGCCTGCACGCCCAGCAG**T**G**C**ATGC
XL095	GCAT**G**C**A**CTGCTGGGCGTGC
XL097	GTCAGAATTCGCTAGCGGTTGCCAGGTTCGCCG (EcoRI, NheI)
XL101	CGCCCTGATCGGCGCC**GCA**CTCGGCGGCATGC
XL102	GCCGCCGAG**TGC**GGCGCCGATCAG
XL136	CTCTTCCAGATCTCCAGCAGG (BglII)
XL137	CTTGATCGAGGA**CGC**GAAGGAGAAC
XL138	CTCCTTC**GCG**TCCTCGATCAAGCAG
XL202	ACCTCCGGATCCACCGGACG (BamHI)
XL203	GCACCGCCCGCAGGG**C**ATCGCCGCCGGCGGCGTAG
XL204	ACGCCGCCGGCGGCGAT**G**CCCTGCGGGCGGTGCACC
XL206	CTGGATCAGGCGGATGG**C**GTCGCCGCCGACCTGG
XL207	CTTCCAGGTCGGCGGCGAC**G**CCATCCGCCTGATCCAG
XL208	ACGTGGATCCTACGCCGACCTTCTCGCC (BamHI)
XL212	ACGTGGATCCGATGTGCTGCTCGGTCTCG (BamHI)
XL213	ACGTGAATTCTCCAGTCGAACGGGGTGGC (EcoRI)

^a^Specified restriction sites are underlined, altered nucleotides are in bold.

### CONSTRUCTION OF OVEREXPRESSION PLASMIDS FOR PROTEIN PURIFICATION

For overexpression of *ambB*, the gene was PCR-amplified from chromosomal DNA of PAO1 using the primer pair XL009/XL010. The 3.8-kb PCR fragment was trimmed with NheI and EcoRI and cloned between the same sites in pET-28a. This generated pME9713 in which the *ambB* gene is fused in-frame to six histidine codons at its 5′ end and expressed under the control of the *T7* promoter. To construct pME10327 (for purification of AmbB_S768A_), a 2.6-kb BamHI-EcoRI fragment with the thiolation site mutation was generated by overlap extension PCR from chromosomal DNA of PAO1 using the primer pairs XL202/XL203 and XL204/XL010. This fragment was then used to replace the corresponding fragment in pME9713.

For construction of the *ambC* expression plasmid pME9718, a 1.1-kb fragment was PCR-amplified from chromosomal DNA of PAO1 with primers XL036 and XL044, trimmed with NdeI and EcoRI, and cloned into pET-27b between the same sites. The *ambD* expression plasmid pME9717 was made in the same way, using primers XL034 and XL042 instead.

Plasmids for *ambE* expression were constructed as follows. The *ambE* gene was PCR-amplified from chromosomal DNA of PAO1 using the primer pair XL007/XL033. The resulting fragment of 6.4 kb was then cloned into pET-27b between the NdeI and NheI restriction sites. This produced pME9714 in which *ambE* is fused in-frame to six histidine codons at its 3′end and expressed from the *T7* promoter. Variants of pME9714 with mutations in the T1 domain (pME10337), the T2 domain (pME10317), and the TE domain (pME10328) were constructed by overlap extension PCR using the primer pairs XL032/XL206 + XL207/XL097 (for pME10337), XL032/XL094 + XL095/XL097 (for pME10317), and XL032/XL102 + XL101/XL097 (for pME10328), respectively. This generated 2.6-kb fragments which were trimmed with BglII and NheI and used to replace the corresponding fragment in pME9714. Plasmid pME10330, which carries mutations in the the A and in the TE domain, was generated in several steps. First, the primer pairs 2302Amut-1/2302Amut-2 and 2302A-mut3/2302A-mut4 were used to generate the codon mutation K1230T. The PCR fragment was then trimmed with NcoI-BglII and used to replace the corresponding fragment in pME9714. The resulting plasmid served as a template in a second overlap extension PCR with XL046/XL137 and XL138/XL36 to generate a fragment specifying the mutations K1230T and D644A. This fragment was then trimmed with PstI and BglII and used to replace the corresponding 2.6-kb fragment in the TE mutant plasmid pME10328.

### MUTANT CONSTRUCTION

Gene replacement mutants of *P. aeruginosa* (**Table 1**) were generated with suicide plasmids as described previously (Ye et al., 1995). These were constructed as follows. To generate the suicide plasmid pME10326 (for generation of *ambB*_T2302G_ specifying AmbB_S768A_), a 2.6-kb fragment was generated from pME9713 by overlap extension PCR using the primer pairs XL202/XL203 and XL204/XL010. This fragment was trimmed with BamHI and EcoRI and cloned into pME3087 between the same sites. For construction of the suicide plasmid pME10338 (to generate *ambE*_T3856G_ specifying AmbE_S1286A_), a 1.1-kb PCR fragment was amplified from pME10337 with the primer pair XL212/XL213. This fragment was trimmed with EcoRI and BamHI and cloned into pME3087. Similarly, a 1.8-kb PCR fragment, amplified from pME10317 with the primer pair XL097/XL208 and trimmed with BamHI and EcoRI, was cloned into pME3087 to obtain the suicide plasmid pME10341 (for construction of *ambE*_T5455G,G5457A_ specifying AmbE_S1819A_). Using pME497 as a helper, the suicide plasmids were mobilized from *E. coli* DH5α into *P. aeruginosa* PAO1 and chromosomally integrated with selection for Tc resistance. Excision of the vector, resulting from a second crossing-over event, was obtained by enrichment for Tc-sensitive cells. Mutant identification was done by PCR and sequence analysis. This generated the mutants PAO6932 (the suicide plasmid used was pME10326), PAO6934 (suicide plasmid pME10338), and PAO6935 (suicide plasmid pME10341).

### DETECTION OF AMB PRODUCTION IN *P. aeruginosa* STRAINS

AMB production of wildtype and mutant strains was assessed using a previously described bioassay, which is based on the growth inhibition of *E. coli* K-12 (Lee et al., 2010).

### PROTEIN PURIFICATION

Hexahistidine-tagged proteins were purified from 800 ml cultures of *E. coli* BL21 (DE3)/pLys (for AmbB and AmbE) or *E. coli* BL21 (DE3; for AmbC, AmbD, and Sfp) carrying the relevant overexpression plasmid (see **Table 1**). Cultures were set up in four Erlenmeyer flasks containing 200 ml of NYB plus Km. These were inoculated with 3 ml of precultures grown overnight in NYB containing Km (for overexpression in *E. coli* BL21 [DE3]) or Km + Cm (for overexpression in *E. coli* BL21 [DE3]/pLys). Growth occurred at 30^∘^C and 180 RPM to an OD_600_ of 0.4–0.5. For purification of AmbB and AmbE, IPTG was then added to a final concentration of 0.5 mM and incubation was continued for another 4 h at 24^∘^C. For purification of AmbC, AmbD, and Sfp, induction was done with 1 mM IPTG and incubation was continued overnight at 20^∘^C.

Cells were harvested by centrifugation (8000 RPM at 4^∘^C for 15 min), washed once with 200 ml of resuspension buffer A (25 mM Tris-HCl pH 7.9, 200 mM NaCl, 5 mM imidazole, and 5 mM β-mercaptoethanol), resuspended in 30 ml of the same buffer, and lysed by two passages through a French pressure cell press (SLM-Amicon). To avoid protein modification and interference with subsequent trypsin digests for MS analyses (see below), no protease inhibitor was used. Cell debris was removed by centrifugation (12’000 RPM at 4^∘^C for 40 min) and proteins were purified from cell lysates by nickel chromatography using Ni-NTA Superflow resin (Qiagen). 3 ml of Ni-NTA slurry was added to the crude cell extract and the sample was rotated at 4^∘^C for 1 h before loading onto a polypropylene column. Column washing occurred with 20 ml of washing buffer (25 mM Tris-HCl pH 7.9, 200 mM NaCl, 30 mM imidazole, and 5 mM β-mercaptoethanol) and proteins were eluted with 10 ml of elution buffer 1 (25 mM Tris-HCl pH 7.9, 200 mM NaCl, 100 mM imidazole, and 5 mM β-mercaptoethanol) followed by 10 ml of elution buffer 2 (25 mM Tris-HCl pH 7.9, 200 mM NaCl, 200 mM imidazole, and 5 mM β-mercaptoethanol). Fractions of 1 ml were collected and analyzed for the presence of the hexahistidine-tagged protein by sodium dodecyl sulfate-polyacrylamide gel electrophoresis (SDS-PAGE). For AmbC and AmbD, EDTA was added to each eluant fraction to a final concentration of 1 mM to chelate metal ions. Fractions with pure (≥90%) hexahistidine-tagged protein were pooled and dialyzed using a regenerated cellulose membrane (3500 molecular weight cutoff, Spectra/Por, Spectrum Laboratories) against 2 lt of dialysis buffer [25 mM Tris-HCl pH 7.9, 200 mM NaCl, 10% glycerol, 0.5 mM β-mercaptoethanol (for AmbB, AmbE, and Sfp) and 25 mM Tris-HCl pH 7.9, 200 mM NaCl, 10% glycerol (for AmbC and AmbD)]. Proteins were subsequently concentrated with Amicon Ultra centrifugal filters with molecular weight cutoffs of 10’000 (for AmbC, AmbD, and Sfp) or 50’000 (for AmbB and AmbE). Concentrated proteins (∼20 mg/ml, as determined by Nanodrop) were flash-frozen in liquid nitrogen and stored in aliquots at -80^∘^C.

### ATP-[^**32**^P]PYROPHOSPHATE EXCHANGE ASSAYS

ATP-[^32^P]pyrophosphate exchange assays were used to demonstrate A-domain substrate specificity. Reactions were carried out in 100 μl volumes containing 75 mM Tris-HCl (pH 7.5), 10 mM MgCl_2_, 0.5 mM tris(2-carboxyethyl)phosphine (TCEP), 5 mM ATP, 1 mM tetrasodium pyrophosphate, 1 mM of amino acid and 20 μl of tetrasodium [^32^P]pyrophosphate (5 mM, 2 μCi/μmol, NEN-Perkin Elmer). Reactions were initiated by the addition of enzyme at 1 μM and incubated at 24^∘^C for 2 h. At appropriate time points, reactions were quenched by the addition of 0.5 ml of charcoal suspension (0.1 M tetrasodium pyrophosphate, 0.35 M perchloric acid, 1.6% (w/v) activated charcoal). Free [^32^P]pyrophosphate was removed by centrifugation and the charcoal pellet was washed twice with wash solution (0.1 M tetrasodium pyrophosphate, 0.35 M perchloric acid). Charcoal-bound radioactivity was measured in a Beckman LS 6500 scintillation counter.

### PHOSPHOPANTETHEINYLATION AND AMINOACYLATION ASSAYS

T-domain loading was assayed using an aminoacylation assay. Apoenzymes (at 2.5 μM) were first incubated in 75 mM Tris-HCl pH 7.5, 5 mM MgCl_2_, 5 mM TCEP, 40 mM NaCl, 0.5 mM CoA with 1.5 μM of purified promiscuous phosphopantetheinyl transferase Sfp at 28^∘^C for 60 min for conversion of the apo- to the holo-form. Subsequently, ^14^C-labeled substrate amino acids were added to a final concentration of 10 μM (L-Ala) or 50 μM (L-Glu) and aminoacylation reactions started with the addition of ATP to a final concentration of 10 mM. At defined time points, samples were drawn and quenched into 800 μl of 10% (v/v) trichloroacetic acid (TCA) and 100 μl of 1 mg/ml BSA. Precipitated proteins were pelleted by centrifugation at 13’000 RPM for 10 min. Protein pellets were washed once with 800 μl 10% TCA and finally dissolved in 250 μl formic acid. ^14^C-bound radioactivity was measured by liquid scintillation counting using a Beckman LS 6500 scintillation counter.

### GENERATION OF ENZYME-ATTACHED AMB SUBSTRATES AND PRECURSOR PEPTIDES FOR MS ANALYSIS

Apoenzymes (at 2.5 μM) were incubated with Sfp as described above to generate their holoforms. After 60 min, the reaction mixture was supplemented with 10 mM ATP and 1 mM of the substrate amino acid(s). For modification of the growing peptide chain, additional enzymes, and potential cofactors were added at the following concentrations: 2.5 μM each of AmbC and AmbD, 1 mM each of *S*-adenosylmethionine (SAM), and α-ketoglutarate (α-KG), and 0.5 mM of ferrous iron (supplied as [NH_4_]_2_Fe[SO_4_]_2_. Samples were incubated at 28^∘^C for another 2 h, supplemented with 10% glycerol, and stored at -80^∘^C until analysis by MS.

For substrate identification, holoenzymes, and ATP were incubated not with a single, defined amino acid but with a pool of all 20 proteinogenic amino acids (each at 0.5 mM final concentration) or with a *P. aeruginosa* metabolome prepared as described previously for *E. coli* (Dorrestein et al., 2006).

### MS ANALYSIS

MS analyses were performed by the Protein Analysis Facility (University of Lausanne). Frozen protein samples were thawed and digested by trypsin using a protocol for short digestion time (Hollenhorst et al., 2010). Briefly, 5 μl of proteins were digested at 30^∘^C for 60 min under agitation with 281.3 ng of modified sequencing-grade porcine trypsin (Promega) at a protein:trypsin ratio of about 10:1 (w/w) in 50 μl of 50 mM ammonium bicarbonate. Digestion was stopped by adding 2 μl of 10% formic acid, and samples were dried by speed-vacuum. Samples were then resuspended in 50 μl of H_2_O:MeCN 97:3 (v/v) + 0.1% formic acid.

The tryptic peptide samples were analyzed by high resolution liquid chromatography coupled with MS (LC-MS/MS) on an Ultimate 3000 RSLCnano HPLC system coupled to a hybrid linear trap LTQ-Orbitrap XL mass spectrometer (Thermo Scientific). Solvents used were (A) H_2_O:MeCN 97:3 (v/v) + 0.1% formic acid and (B) H_2_O:MeCN 20:80 (v/v) + 0.1% formic acid. A volume of 5 μl of sample was loaded onto a trapping microcolumn Acclaim PepMap 100 C18 (2 cm × 100 μm, Dionex) in H_2_O:MeCN 97:3 (v/v) + 0.1 formic acid at a flow rate of 3.5 μl/min. Peptides were then eluted and separated on a reversed-phase Acclaim PepMap RSLC C18 nanocolumn (75 μm ID × 15 cm, 2 μm, Dionex) or a Nikkyo C18 nanocolumn (75 μm ID × 15 cm, 3 μm, Nikkyo Technos) with a 95 min gradient, at a flow rate of 300 nl/min. In data-dependent acquisition controlled by Xcalibur 2.0 software (Thermo Scientific), the eight most intense precursor ions detected in the full MS survey performed in the Orbitrap (range 350–1700 mass/charge (*m/z*) ratio, resolution 60 000 at *m/z* 400) were selected for fragmentation, and fragment ions were analyzed in the ion trap. MS^2^ was triggered by a minimum signal threshold of 10’000 counts and carried out at relative collision energy of 35% (CID), with an isolation width of 4.0 amu. Only precursors with a charge higher than one were selected for fragmentation and the *m/z* of fragmented precursors was then dynamically excluded from any selection during 60 s. MS^3^ analyses were further carried out for specific detection of phosphopantetheinylated (PPant) peptides as described by Meier et al. (2011). Briefly, following MS^2^ of the most intense precursor ion from an inclusion list, the two most intense fragment ions were selected for MS^3^ fragmentation in the ion trap, if their mass was present in an inclusion list of pantetheine fragments and above a signal threshold of 100 counts in the MS/MS spectrum.

MS/MS spectra were analyzed using Mascot 2.4 (Matrix Science, London, UK). Mascot driven search was set up against a custom-built database containing the *P. aeruginosa* sequences of wild-type and mutated AmbE and AmbB, and the sequences of usual contaminants (enzymes, keratins, etc). Trypsin (semi-specific cleavage at K, R, not before P) was used as the enzyme digestion definition allowing up to two missed cleavages. Mascot was searched with a parent ion tolerance of 10 ppm and a fragment ion mass tolerance of 0.5 Da. Acetylation at protein N-terminal, deamidation of asparagine, and glutamine, oxidation of methionine, and phosphopantetheine addition to serine were specified as variable modifications.

### BIOINFORMATICS ANALYSIS

DNA and protein sequences were obtained from the *P.* genome database (Winsor et al., 2011) and the protein and genome databases available on the NCBI website ^1^. Protein sequences were analyzed on the polyketide synthase/NRPS analysis website (Bachmann and Ravel, 2009) and searched against the Conserved Domain Database using the CD-search algorithm on the NCBI website (Marchler-Bauer and Bryant, 2004). C-domain phylogenetic analysis was performed using the ClustalW algorithm (Thompson et al., 1994) available on the server of the Kyoto University Bioinformatics Center ^2^. Results were displayed as an unrooted N-J phylogenetic tree (Saitou and Nei, 1987).

## RESULTS

### DOMAIN ARCHITECTURE OF AmbB AND AmbE

Sequence inspection of AmbB and AmbE suggested that these enzymes are NPRSs that generate a AMB precursor peptide by a thiotemplate mechanism (Stein et al., 1996). NRPSs can be dissected into modules, each module being responsible for the incorporation of one amino acid or carboxy acid into the growing peptide chain. In turn, each module consists of domains, which are enzymatic units that catalyze the stepwise addition and modification of the amino/carboxy acid. In some cases, the biosynthesis of a non-ribosomal peptide (NRP) can be predicted from the analysis of these domains provided that biosynthesis follows the colinearity rule (Marahiel et al., 1997). Bioinformatics analysis showed that the AmbB protein is composed of three domains, which are responsible for adenylation (A domain) of a substrate amino acid, for loading of this amino acid onto a phosphopantetheine arm attached at a conserved serine (T domain), and for condensation (C domain) of this amino acid with a second amino acid bound at the T domain of another module (**Figure 1**). The modular structure of AmbE is more complex and consists of an A domain, two T domains (T1 and T2), a C domain (indicated as C^∗^ in **Figure 1**), a domain for methylation (MT domain) and a domain for thioester cleavage (TE domain). Moreover, it cannot be ruled out that an additional domain of unknown function may be located at the N-term of AmbE (**Figure 1**) given that this consists of a stretch of 461 amino acids. However, no similar domain architectures have been identified in this region using the Conserved Domains algorithm. Phylogenetic analyses of the C^∗^ domain suggest a new or additional function of this C domain, as it does not group with previously known L2L, D2L, epimerization, heterocyclization, dual condensation/epimerization, or starter C domains (Figure S1). The C^∗^ domain rather groups with another atypical C domain found in the McyA protein of *Microcystis aeruginosa* (Tillet et al., 2000). However, the biochemical function of McyA has not been studied experimentally. We conclude from these analyses that the two peptide synthetases AmbB and AmbE likely generate a tripeptide. This peptide is suspected to undergo a series of modification steps carried out by tailoring domains of AmbE (MT domain, N-term domain, C^∗^ domain), and by the hydroxylases AmbC and AmbD. Finally, the tripeptide would be released from the thiotemplate by thioester cleavage (TE domain).

### OVEREXPRESSION AND PURIFICATION OF THE AMB BIOSYNTHESIS PROTEINS AmbB, AmbC, AmbD, AND AmbE

**FIGURE 2:**
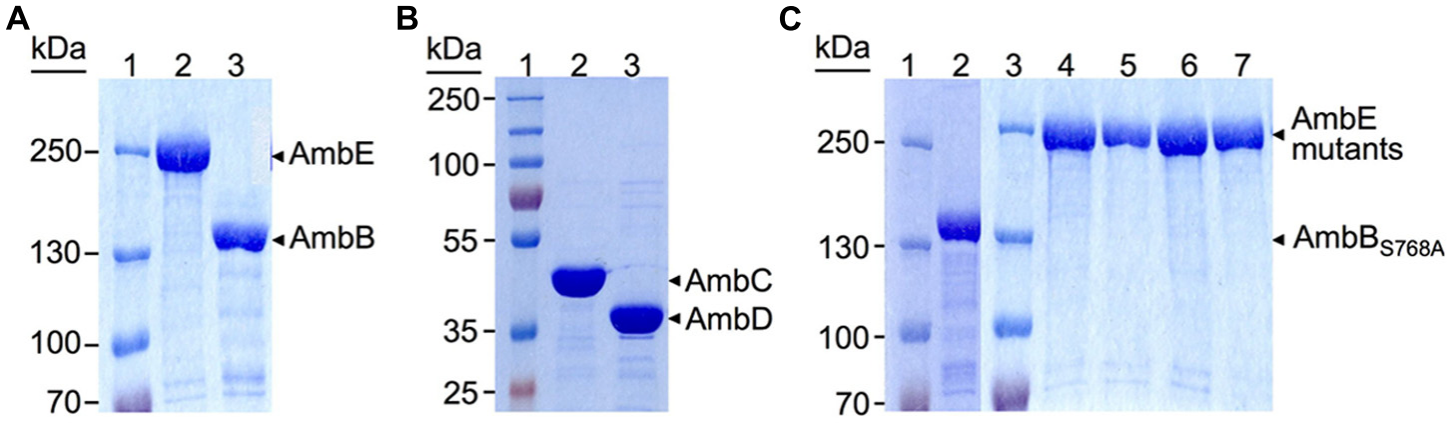
**Sodium dodecyl sulfate-polyacrylamide gel electrophoresis (SDS-PAGE) analysis of hexahistidine-tagged proteins purified by Ni-NTA chromatography. (A)** 6% Separating gel with protein ladder (lane 1), and AmbE (231.5 KDa, lane 2) and AmbB (137 KDa, lane 3) purified from BL21[DE3]/pLys carrying pME9714 and pME9713, respectively. **(B)** 10% Separating gel with protein ladder (lane 1), and AmbC (41.5 KDa, lane 2), and AmbD (39.5 KDa, lane 3) purified from BL21[DE3] carrying pME9718 and pME9717, respectively. **(C)** 6% Separating gels of purified mutant proteins. Lanes 1 and 3, protein ladders; lane 2, AmbB_S768A_ expressed from pME10327; lane 4, AmbE_D644A,K1230T,S1958A_ expressed from pME10330; lane 5, AmbE_S1958A_ expressed from pME10328; lane 6, AmbE_S1819A_ expressed from pME10317; lane 7, AmbE_S1286A_ expressed from pME10337. All proteins were purified from BL21[DE3]/pLys carrying the respective plasmid.

To study AMB biosynthesis with purified enzymes *in vitro*, the *ambBCDE* genes (and their mutated derivatives, see below) were cloned under the control of the *T7* promoter in pET-28a or pET-27b (**Table 1**). Proteins were purified from *E. coli* BL21(DE3; for AmbC and AmbD) and BL21(DE3)/pLys (for AmbB and AmbE) with a hexahistidine tag at their N-terminus (AmbB) or C-terminus (AmbC, AmbD, and AmbE). According to analysis on SDS-PAGE gels all proteins were ≥90% pure (**Figure 2**).

### IDENTIFICATION OF L-ALA AS THE AMINO ACID SUBSTRATE OF AmbB

**FIGURE 3:**
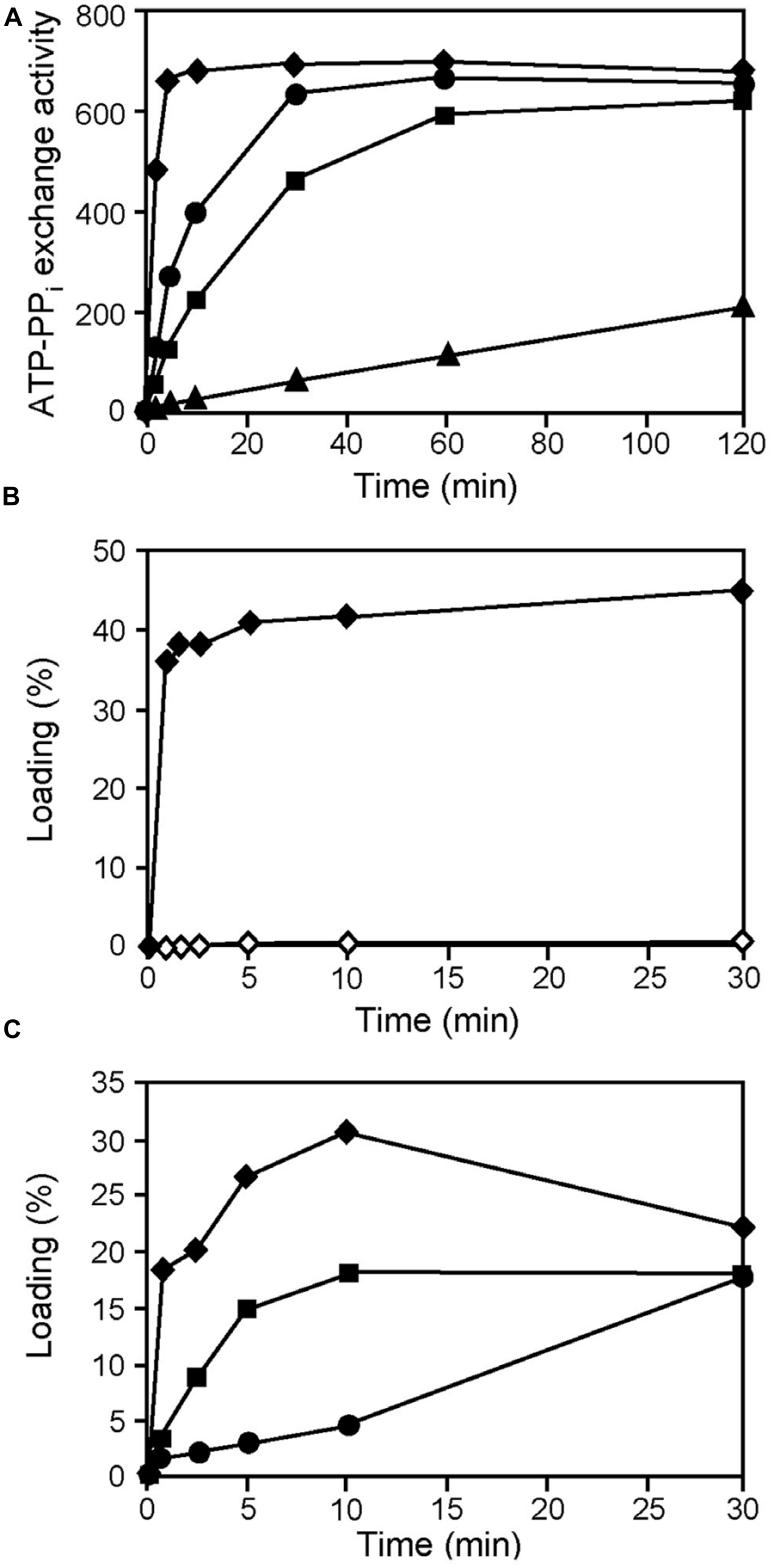
**Identification of L-Ala as the preferred amino acid substrate activated by and loaded onto AmbB. (A)** AmbB activation of L-Ala (diamonds), D-Ala (triangles), Gly (rectangles), and L-Ser (circles). AmbB was incubated sequentially with each amino acid in an ATP-[^32^P] pyrophosphate exchange assay. At different time points, generated ATP (formed by the reverse reaction of the AmbB A domain in the presence of [^32^P] pyrophosphate) was bound and counted. ATP-PP_i_ exchange activity was determined for the full course of the assay and is expressed as the amount of conversion (one being 100% conversion) multiplied by the molar ratio of PP_i_ to AmbB. **(B)** Loading of ^14^C-L-Ala onto AmbB (filled diamonds) and AmbB_S768A_ (empty diamonds). Proteins were incubated with radiolabeled L-Ala in an aminoacylation assay. At several time points, proteins (with bound amino acids) were precipitated and radioactivity was counted. Percentage of loading was determined using the molar ratio of bound radioactivity to the amount of protein in the assay. **(C)** Loading of L-Ala, Gly, and L-Ser onto AmbB. AmbB was sequentially incubated with ^14^C-L-Ala (diamonds), ^14^C-Gly (rectangles), and ^14^C-L-Ser (circles) in an aminoacylation assay. At several time points, AmbB (with bound amino acids) was precipitated and radioactivity was counted. Percentage of loading was determined using the molar ratio of bound radioactivity to the amount of protein in the assay.

Based on the presence of key residues found in the A-domains of peptide synthetases, it is often possible to predict their amino acid specificity (Stachelhaus et al., 1998). Predictions of A domain specificity in AmbB favors the activation of L-Ala but certain residues of the binding pocket have also been found in D-alanine (D-Ala) activating A domains of other peptide synthetases. We therefore tested which of the two isomers would be activated by AmbB using an ATP-[^32^P]pyrophosphate exchange reaction. In addition, we also investigated whether other small amino acids such as glycine (Gly) or L-serine (L-Ser) would be activated. As shown in **Figure 3A**, AmbB activated preferentially L-Ala, giving an exchange activity of 653 at 5 min already. Hower, L-Ser, Gly, and D-Ala were activated as well, albeit to a lower extent. Exchange activities measured at 5 min were 270, 128, and 114 for L-Ser, Gly, and D-Ala, respectively (**Figure 3A**). To further specifiy the amino acid substrate, we next investigated T domain loading of AmbB. For this, proteins were incubated first with CoA and the promiscuous phosphopantetheinyltransferase Sfp of *Bacillus subtilis* for phosphopantetheinylation of the T domain. Loading was then monitored by measuring the incorporation of ^14^C-labeled amino acids into AmbB (and AmbB_S768A_, see below) by TCA precipitation and liquid scintillation counting. As shown in **Figure 3B**, loading of AmbB with ^14^C-L-Ala occurred very quickly, reaching a maximum of 35–40% after 1 min only. By contrast, no loading was observed with AmbB_S768A_. In this protein, the active site Ser in the conserved thiolation motif LGG(H/D)S(L/I; Stein et al., 1996) was altered, thus preventing cofactor attachment and hence loading of L-Ala. The importance of Ser 768 of AmbB was also tested *in vivo*. As expected, a *P. aeruginosa* strain (PAO6932) with a chromosomally mutated *ambB* gene (specifying AmbB_S768A_) lost the ability to make AMB (Figure S2).

As ATP-[^32^P]pyrophosphate exchange reactions had revealed some activation of Gly and L-Ser by AmbB (**Figure 3A**), we tested whether these amino acids would be loaded as well. Indeed, loading was observed for both of them but to a lesser extent than with L-Ala (**Figure 3C**). Taken together, we conclude that the preferred substrate of AmbB is L-Ala and that this amino acid is loaded onto the phosphopantetheine arm attached at Ser 768.

### ACTIVATION AND LOADING OF L-GLU BY AmbE

**FIGURE 4:**
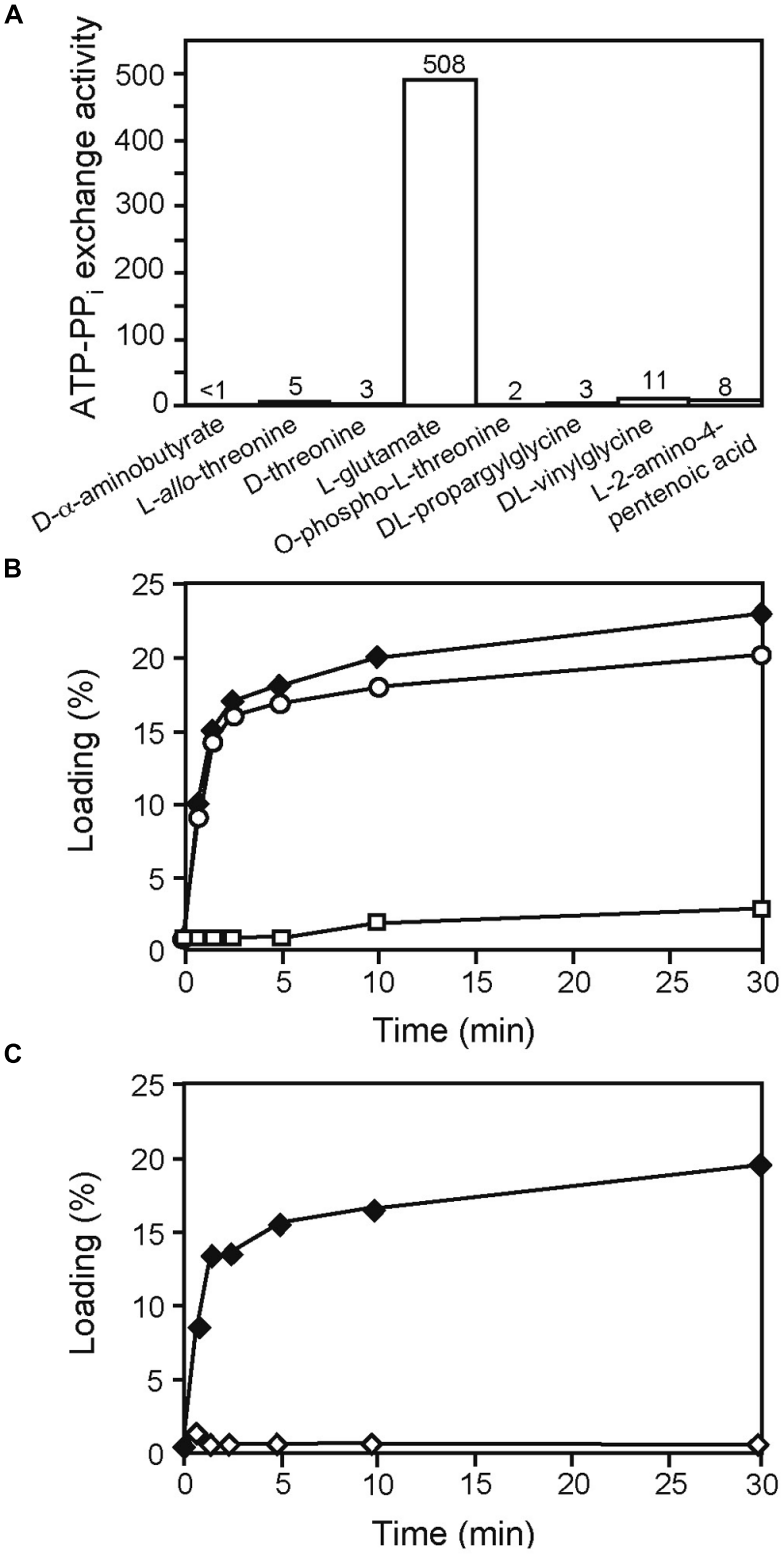
**Identification of L-Glu as the amino acid substrate activated by AmbE and loaded onto its T1 domain. (A)** End-point ATP-[^32^P] pyrophosphate exchange assay to screen potential AmbE substrates. At 120 min, generated ATP (formed by the reverse reaction of the A domain of AmbE in the presence of [^32^P] pyrophosphate) was bound and counted. ATP-PP_i_ exchange activity is expressed as the amount of conversion (one being 100% conversion) multiplied by the molar ratio of PP_i_ to AmbE. **(B)** Loading of ^14^C-L-Glu onto AmbE (filled diamonds), AmbE_S1286A_ (empty rectangles), and AmbE_S1819A_ (empty circles). Proteins were incubated with the radiolabeled amino acid in an aminoacylation assay. At several time points, proteins (with bound amino acids) were precipitated and radioactivity was counted. Percentage of loading was determined using the molar ratio of bound radioactivity to the amount of protein in the assay. **(C)** Mutations in the A domain of AmbE abolish protein loading with L-Glu. AmbE_S1958A_ (filled diamonds), and AmbE_D644A,K1230T,S1958A_ (empty diamonds) were incubated with ^14^C-L-Glu in an aminoacylation assay. At several time points, proteins (with bound amino acids) were precipitated and radioactivity was counted. Percentage of loading was determined using the molar ratio of bound radioactivity to the amount of protein in the assay. Note that the mutation of the active site Ser (S1958) of the TE domain does not interfere with loading of L-Glu. By contrast, amino acid alterations in the A domain (D644A and K1230T) abolished loading entirely.

Unlike with AmbB, an exact prediction of the amino acid substrate for AmbE was not evident from the Stachelhaus motif. As this motif predicted a polar residue, we first considered L-α-aminobutyrate, L-homoserine, L-aspartate, and L-threonine as AMB precursor amino acids. However, none of these amino acids was activated in an ATP-[^32^P]pyrophospate exchange assay (data not shown). We next tested D-α-aminobutyrate, L-*allo*-threonine, D-threonine, L-Glu, *O*-phospho-L-threonine, DL-propargylglycine, DL-vinylglycine, and L-2-amino-4-pentenoic acid. As shown in **Figure 4A**, strong activation of L-Glu was observed. We therefore tested whether L-Glu would be loaded onto AmbE and found that this was indeed the case (**Figure 4B**). By contrast, no loading of L-Glu was observed with the mutant AmbE_D644A,K1230T_ affected in the adenylation domain (**Figure 4C**). To investigate on which of the two T domains L-Glu would be loaded, we carried out loading assays with AmbE_S1286A_ and AmbE_S1819A_, two mutant proteins affected in the active site Ser residues of the T1 and T2 domains, respectively. As shown in **Figure 4B**, loading was observed with AmbE_S1819A_ but not with AmbE_S1286A_. To verify that both Ser residues are important for cofactor attachment, we mutated the respective codons in the chromosome of *P. aeruginosa*, giving PAO6934 and PAO6935, respectively. As expected, both strains had lost the ability to make AMB (Figure S2). We thus conclude that the A domain of AmbE activates L-Glu, which is then loaded onto T1 but not onto T2.

### AmbB-DEPENDENT LOADING OF L-ALA ONTO THE T2 DOMAIN OF AmbE

**FIGURE 5:**
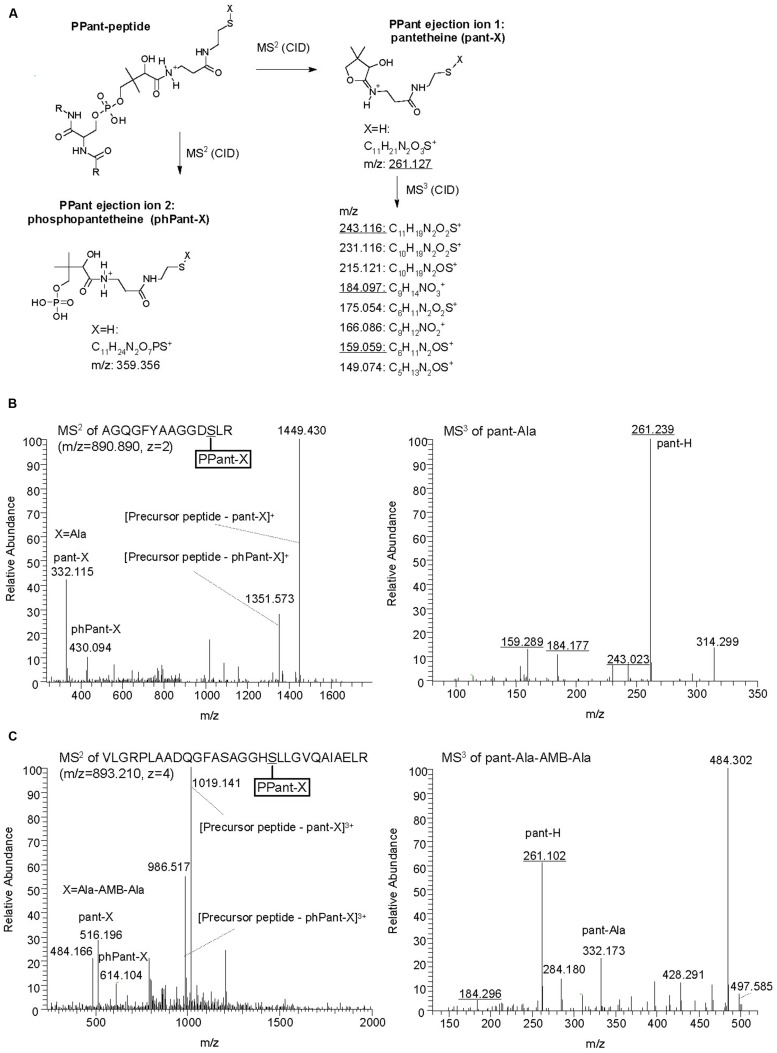
**Identification of AmbB/AmbE-bound substrates and pathway intermediates by phosphopantetheinyl elimination reactions. (A)** Structure of PPant ejection ions after MS^2^ fragmentation and pantetheine-specific MS^3^ pattern (adapted from Meluzzi et al., 2008; Meier et al., 2011). Diagnostic m/z values obtained in B and C are underlined. **(B)** Identification of Ala on AmbB by characteristic MS^2^ and MS^3^ spectra. AmbB and AmbE (at 2.5 μM; converted to their holo-forms by Sfp) were incubated with 0.5 mM of all 20 proteinogenic amino acids before trypsin digestion and analysis by MS. **(C)** Identification of Ala-AMB-Ala on AmbE by characteristic MS^2^ and MS^3^ spectra. The assay contained AmbB, AmbC, AmbD, and AmbE_S1958A_ (at 2.5 μM; the two NRPSs were converted to their holo-forms by Sfp), L-Ala and L-Glu (at 1 mM), as well as the cofactors SAM, α–KG (at 1 mM) and ferrous iron (at 0.5 mM). After incubation, the sample was treated with trypsin and analyzed by MS.

The identity of the substrate loaded onto the T2 domain of AmbE was investigated using MS-based activity screening (Dorrestein et al., 2006; Meier et al., 2011). This method allows substrate identification based on the mass changes that take place during the acylation of the T domain-attached phosphopantetheine arm (**Figure 5A**). AmbE, together with AmbB (which was included in the same experiment as an internal control), was presented either with a mixture of all 20 natural amino acids or with a *P. aeruginosa* metabolome before being digested with trypsin and analyzed by LC-MS/MS (see Section “Materials and Methods” for experimental details). Similar results were obtained in both cases. The phosphopantetheinylated AmbB peptide AGQGFYAAGGDSLR presented a mass shift of +71.04 indicative of Ala loading onto the T site of AmbB (**Figure 5B**; **Table 3**). In agreement with the protein’s previously observed substrate promiscuity, the same peptide also showed mass shifts characteristic for loading of Gly (57.02; Figure S3A; **Table 3**) and Ser (87.03; Figure S3B; **Table 3**). To identify the substrates loaded onto AmbE, mass shifts of the phosphopantetheinylated peptides RPAIGVSDNFFQVGGDSIR (T1 domain) and VLGRPLAADQGFASAGGHSLLGVQAIAELR (T2 domain) were analyzed. As expected, we found a Glu-specific mass shift of +129.04 for the T1-specific peptide (Figure S4A; **Table 3**). For the T2-specific peptide we detected a mass shift of +71.04 (Figure S4B; **Table 3**), indicating that the substrate amino acid loaded at the second T domain of AmbE could be Ala.

**Table 3:** AmbB and AmbE peptides with AMB substrates and pathway intermediates loaded via phosphopantetheine.

Peptide^a^	Substrate or pathway intermediate X loaded (theoretical mass)	Theoretical m/z (z) (monoisotopic mass)	Observed m/z (Δm ppm)	m/z pant-X	Amb enzymes required for detection of attached compound
AGQGFYAAGGDSLR (AmbB, T domain)	Gly (57.021) Ala (71.037) Ser (87.032)	883.883 (2) 890.891 (2) 898.889 (2)	883.881 (-2.3) 890.890 (-1.1) 898.886 (-3.3)	318.1 332.2 348.2	AmbB AmbB AmbB

RPAIGVSDNFFQVGGDSIR (AmbE, T1 domain)	Glu (129.043) Glu-Ala (200.080)	835.392 (3) 859.071 (3)	835.393 (1.2) 859.072 (1.2)	390.2 461.2	AmbE/AmbE_S1819A_^b^ AmbB+AmbE_S1819A_^b^

VLGRPLAADQGFASAGGHSLLGVQAIAELR (AmbE, T2 domain)	Ala (71.037) Ala-AMB-Ala (255.121) Ala-Glu-Ala (271.117)	847.192 (4) 893.212 (4) 897.211 (4)	847.189 (-3.5) 893.210 (-2.2) 897.213 (-2.2)	332.2 516.2 532.2	AmbB+AmbE/AmbE_S1819A_^b^ AmbB+AmbC+AmbD+AmbE_S1819A_^b^ AmbB+AmbE_S1819A_^b^

^a^Peptides were generated by trypsin digestion of reaction mixtures containing the indicated Amb enzymes. ^b^For increased detection of enzyme-attached compounds, the thioesterase-negative mutant protein AmbE_*S1819A*_ was used in certain experiments. The Ser residues carrying phosphopantetheine-linked substrates and pathway intermediates are underlined.

**FIGURE 6:**
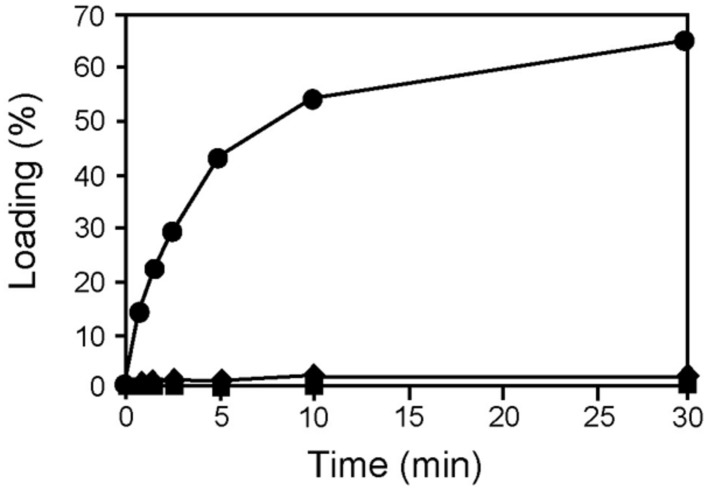
**AmbB-dependent loading of L-Ala onto the T2 domain of AmbE.** AmbE (filled squares), AmbE + AmbB_S768A_ (filled circles), and AmbE_S1819A_+ AmbB_S768A_ (filled diamonds) were incubated with ^14^C-L-Ala in an aminoacylation assay. At several time points, proteins (with bound amino acids) were precipitated and radioactivity was counted. Percentage of loading was determined using the molar ratio of bound radioactivity to the amount of AmbE/AmbE_S1819A_ in the assay.

The potential identity of the T2-attached substrate with Ala suggested that this amino acid may have been loaded *in trans* from AmbB. This hypothesis was tested using the AmbB_S768A_ mutant protein which is able to activate L-Ala but cannot load it onto its own T domain (see **Figure 3B**). As shown in **Figure 6**, the presence of AmbB_S768A_ in an aminoacylation assay of AmbE allowed ^14^C-L-Ala to be loaded, reaching a maximum of over 60% labeled protein after 30 min. By contrast, no loading was observed in the absence of AmbB_S768A_ or when AmbE was replaced by the T2 domain mutant AmbE_S1819A_. We thus conclude that AmbB loads L-Ala *in cis* onto its own T domain and *in trans* onto the T2 domain of AmbE.

### ASSEMBLY OF A TRIPEPTIDE AMB PRECURSOR ON AmbE

The identification of the substrate amino acids activated by and loaded onto AmbB and AmbE suggested that these proteins generate an L-Ala-L-Glu-L-Ala tripeptide. We speculated that the central L-Glu would be the amino acid to be converted to AMB by AmbC, AmbD, and tailoring domains of AmbE, while the two flanking L-Ala residues may function as protective groups during these modifications. Indeed, LC-MS/MS analysis of *in vitro* reactions carried out with different enzyme combinations, revealed several characteristic mass shifts of the phosphopantetheinylated T1 and T2 peptides of AmbE (**Table 3**). A reaction mixture containing AmbB, AmbE_S1958A_ (a thioesterase-negative AmbE variant used to stabilize enzyme-attached AMB precursor peptides), L-Ala and L-Glu, generated not only the previously observed mass shifts of the phosphopantetheinylated amino acid substrates [+129.04 for Glu and +71.04 for Ala; data not shown), but also mass shifts of +200.08 (at T1) and +271.12 (at T2; Figures S5A,B]. These masses are identical with those expected for a T1-attached Glu-Ala dipeptide and for a T2-attached Ala-Glu-Ala tripeptide, respectively. When the two oxygenases (AmbC and AmbD), their cofactors (α-ketoglutarate and ferrous iron), and SAM (cofactor for the MT domain of AmbE) were included in the assay, the mass shifts of these pathway intermediates were no longer detected. Instead, a new mass shift of +255.12 was identified for the T2-specific peptide (**Figure 5C**). This mass corresponds to that expected for an Ala-AMB-Ala tripeptide.

## DISCUSSION

**FIGURE 7:**
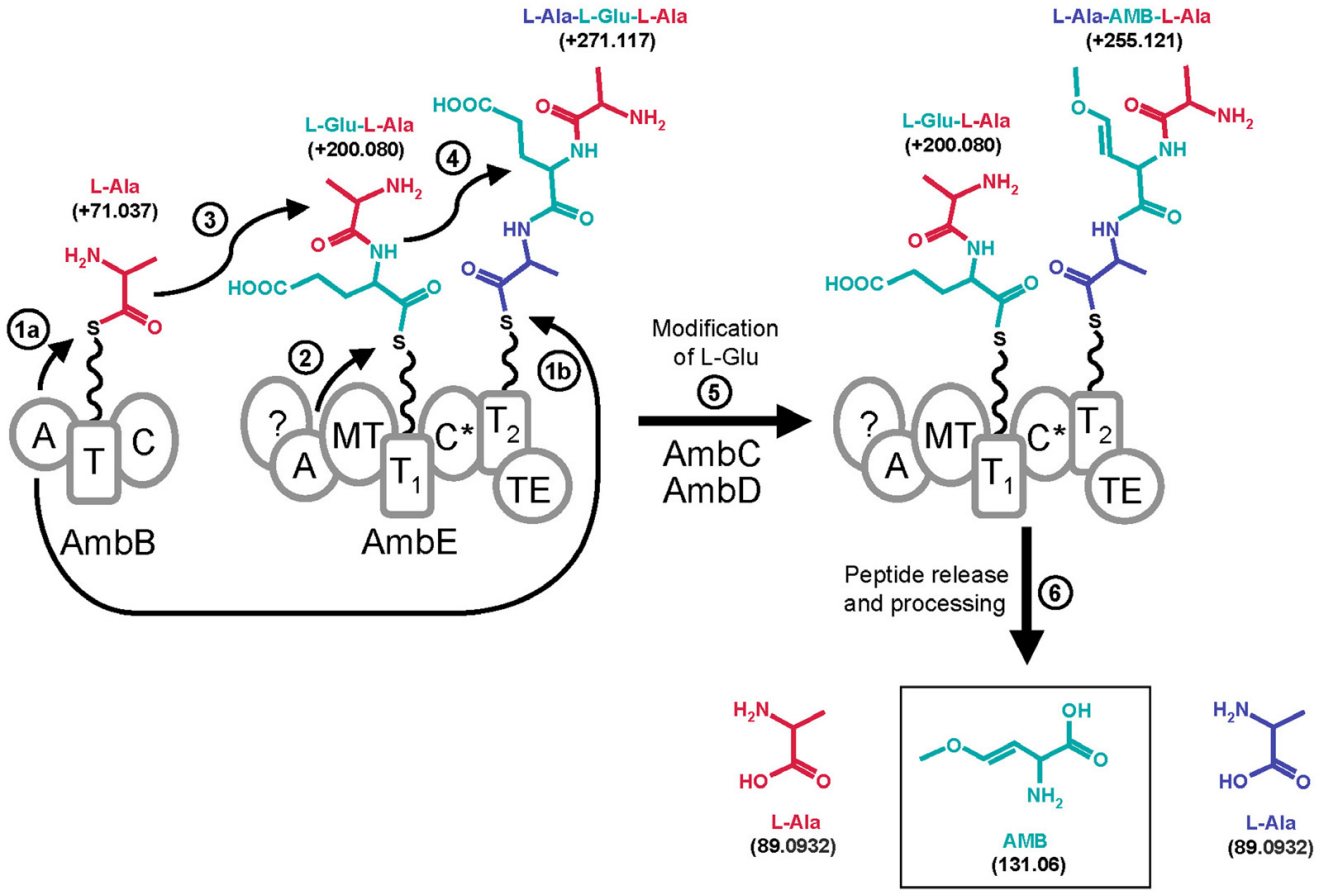
**Proposed model for AMB biosynthesis with indication of mass shifts (in Da) of tethered molecules.** The A domain of AmbB activates L-Ala (residue shown in red) and loads it onto its own T domain (step 1a) and onto the T2 domain of AmbE (step 1b). The A domain of AmbE activates L-Glu (residue shown in blue) and loads it onto its T1 domain (step 2). Then, the C domain of AmbB or the C* domain of AmbE would condense L-Ala with L-Glu onto the T1 domain of AmbE forming the L-Glu-L-Ala dipeptide (step 3). This would subsequently be condensed with L-Ala on the T2 domain of AmbE to give the tripeptide L-Ala-L-Glu-L-Ala (step 4). Modification of the central L-Glu by action of AmbC, AmbD, the MT domain of AmbE, and eventually by the C* domain and/or by the N-term of AmbE, would give rise to the tripeptide L-Ala-AMB-L-Ala (step 5). This is believed to be the final peptide released from AmbE by action of the TE domain (step 6). Finally, the flanking L-Ala residues are removed by a processing step to yield AMB (step 6).

In this work we have demonstrated that L-Ala and L-Glu are the amino acid substrates for AMB production. The identity of these building blocks was demonstrated with classical aminoacylation assays using radiolabeled amino acid substrates and also with a MS-based approach which detected these amino acids bound via phosphopantetheinyl arms to the T domains of AmbB and AmbE (see **Figure 7**). Specifically, we detected L-Ala bound to the T domain of AmbB (**Figures 3** and **5B**) and to the T2 domain of AmbE (**Figures 6** and S4B) while L-Glu was bound to the thiolation domain T1 of AmbE (**Figures 4B** and S4A). However, AmbE specifies a single adenylation domain, which was predicted to activate a polar amino acid such as L-Glu. Indeed, L-Glu was no longer loaded onto T1 when this domain was mutated (**Figure 4C**), most likely because the mutations had interfered with L-Glu activation. How is L-Ala bound at the AmbE’s T2 site activated? As L-Ala was identified as the substrate activated by and loaded onto AmbB we hypothesized that AmbB might also be responsible for loading the T2 site of AmbE. Using an AmbB mutant unable to load its own T site we demonstrated that this was indeed the case (**Figure 6**).

The identification of three amino acids as the building blocks required for AMB generation is remarkable as AMB has not been found as a tripeptide but as a single non-proteinogenic amino acid with a molecular weight of 131.06 Da (**Figure 7**). To further investigate this novel biosynthesis we searched for pathway intermediates attached at the thiolation sites of AmbE and found a Glu-Ala dipeptide and an Ala-Glu-Ala tripeptide at T1 and T2, respectively (Figure S5). These compounds were no longer detected when the assay contained, in addition, the oxygenases AmbC and AmbD together with all cofactors. Instead, large amounts of an Ala-AMB-Ala tripeptide attached at T2 were detected (**Figure 5C**). Based on these results we propose a biosynthetic pathway for AMB production which proceeds via the generation of a tripeptide precursor. According to this model (**Figure 7**), the A domain of AmbB activates L-Ala which is loaded onto the protein’s own T domain and onto the T2 domain of AmbE. The A domain of AmbE activates L-Glu, which is loaded onto the T1 domain. The C domain of AmbB then condenses L-Ala and L-Glu to form the L-Glu-L-Ala dipeptide at T1 of AmbE. This dipeptide is subsequently condensed, probably by the C^∗^ domain, with the L-Ala residue attached at T2 to give the L-Ala-L-Glu-L-Ala tripetide at T2. The central amino acid, L-Glu, would then undergo a series of modifications to be converted into AMB while the two flanking L-Ala residues remain in place. The modifications of L-Glu would be carried out by AmbC, AmbD, and tailoring domains of AmbE (**Figure 1**). The order and the timing of these modifications is currently unknown and we cannot exclude at this point that some modifications of L-Glu may occur even before condensation with the two L-Ala residues, as the unmodified di- and tripeptides (L-Ala-L-Glu and L-Ala-L-Glu-L-Ala) were observed only in the absence of AmbC and AmbD. Finally, the L-Ala-AMB-L-Ala tripeptide is released by thioester cleavage (TE domain of AmbE) and processed to give AMB and two molecules of L-Ala.

What is the role of the two flanking L-Ala residues during biosynthesis of AMB? As previously mentioned, AMB inhibits PLP-dependent enzymes (Berkowitz et al., 2006) and as such could act as a growth inhibitor in the producer as well. We thus speculate that the two flanking L-Ala residues could not only act as protective groups during the conversion of L-Glu to AMB, but also have the additional function of preventing AMB toxicity during biosynthesis. It is not yet clear how processing of the Ala-AMB-Ala tripeptide occurs. We believe that the processing peptidase is encoded by *P. aeruginosa* itself as free AMB is readily detected in *P. aeruginosa* culture supernatants (Scannell et al., 1972; Lee et al., 2010). However, we cannot exclude that the Ala-AMB-Ala peptide is also excreted by *P. aeruginosa*, taken up by susceptible neighbors and subjected to peptidase/amidase action to liberate the toxin. Such a “Trojan Horse” mechanism has been described previously for other bacterial toxins such as the herbicide phosphinothricin (PT), which is produced non-ribosomally by *Streptomyces viridochromogens* as an PT-Ala-Ala tripeptide (Metcalf and van der Donk, 2009).

Secretion of AMB (and/or its precursor tripeptide) is expected to be carried out by AmbA, a member of the LysE transporter family (Lee et al., 2010). However given that LysE is an amino acid transporter (Vrljic et al., 1996), there is a higher likelihood that AmbA exports AMB. We speculate that AmbA could also be crucial for resistance of the producer bacterium when free AMB re-enters the cytoplasm. As for AMB import into *P. aeruginosa*, it is not clear which transporter is involved, but it is interesting to note that in *Staphylococcus aureus* a mutation in a D-methionine transport system rendered this bacterium resistant toward AMB (our unpublished observations).

AMB is the first oxyvinylglycine made from L-Glu. In other cases, e.g., 4-AVG (Fernández et al., 2004), 4-FVG (Halgren et al., 2013), and rhizobitoxine (Mitchell and Coddington, 1991; Yasuta et al., 2001), the starting amino acid appears to be homoserine. Moreover, there is no evidence that these compounds are made by a thiotemplate mechanism, or processed from a precursor peptide, suggesting that there is significant diversity in the biosynthesis of oxyvinylglycines.

In summary, we have elucidated here the first steps of the AMB biosynthetic pathway. Clearly, these data confirm our previous results, which showed that the *amb* gene cluster is responsible for the biosynthesis of AMB (Lee et al., 2010, 2013a) and does not specify the quorum-sensing molecule IQS [2-(2-hydroxyphenyl)-thiazole-4-carbaldehyde], as reported by the group of Lian-Hui Zhang (Lee et al., 2013b). The chemical structure of IQS indicates that this compound may be assembled from salicylate and cysteine. However, neither of the two peptide synthetases encoded by the *amb* gene cluster present adenylation domains with a specificity for these substrates and the biochemical assays performed in this work show that AmbB is loaded with alanine while AmbE is loaded with glutamate and alanine. It is thus highly implausible that IQS is specified by the *amb* gene cluster. Unfortunately, the authors of the IQS publication did not verify which molecule was produced in an *E. coli* strain overexpressing *ambBCDE*. Moreover, their biotest used to measure AMB production was not performed as should be (Lee et al., 2010) which caused a misinterpretation of the results. As proposed recently by Ye et al. (2014), IQS – which is actually aeruginaldehyde – seems instead to be a byproduct produced from salicylate coupled to the first moiety of cysteine during biosynthesis of the enantiomeric siderophores pyochelin and enantiopyochelin in *P. aeruginosa* and *P. fluorescens*, respectively (reviewed by Youard et al., 2011).

Many questions concerning the biosynthesis and transport of AMB remain open. It will be fascinating to dissect the function of the tailoring enzymes, to determine the order in which the modifications of L-Glu occur, to identify the processing enzyme, and to investigate the role of AmbA in AMB export and immunity.

## Conflict of Interest Statement

The authors declare that the research was conducted in the absence of any commercial or financial relationships that could be construed as a potential conflict of interest.

## Footnotes

1 http://www.ncbi.nlm.nih.gov

2 http://www.genome.jp/tools/clustalw/
