# Supplementary material for: The Pseudomonas aeruginosa antimetabolite L -2-amino-4-methoxy-trans-3-butenoic acid (AMB) is made from glutamate and two alanine residues via a thiotemplate-linked tripeptide precursor
Source: Front Microbiol. 2015 Mar 12;6:170. doi: 10.3389/fmicb.2015.00170 (PMC4357302; doi:10.3389/fmicb.2015.00170)
Supplement: Supplementary file 1 [file data_sheet_1.pdf]

Supplementary Material

The *Pseudomonas aeruginosa* antimetabolite L-2-amino-4-methoxy-*trans*-3-butenoic acid (AMB) is made from glutamate and two alanine residues via a thiotemplate-linked tripeptide precursor

N. Rojas Murcia[1#], X. Lee[1#], P. Waridel[2], A. Maspoli[1], H. J. Imker[3†], T. Chai[1], C. T. Walsh[3] and C. Reimmann[1*]

[1]Department of Fundamental Microbiology, University of Lausanne, Switzerland
[2]Protein Analysis Facility, University of Lausanne, Switzerland
[3]Department of Biological Chemistry & Molecular Pharmacology, Harvard Medical School, Boston, Massachusetts, USA

#These authors contributed equally

[†]Current address: Research Data Service, University of Illinois at Urbana-Champaign, Urbana, Illinois, USA

Running title: *AMB biosynthesis*

*Correspondence: C. Reimmann, Department of Fundamental Microbiology, University of Lausanne, Biophore Building, Quartier UNIL-Sorge, CH-1015 Lausanne, Switzerland. cornelia.reimmann@unil.ch

Keywords: *Pseudomonas*, toxin, oxyvinylglycine, secondary metabolite, thiotemplate

FIGURE S1. Phylogenetic analysis of the C* domain from AmbE.

FIGURE S2. Biotest for AMB production by different *P. aeruginosa* strains.

FIGURE S3. Identification of AmbB-bound Gly (A) and Ser (B) by phosphopantetheinyl elimination reactions.

FIGURE S4. Identification of the amino acid substrates bound to the T1 and T2 domains of AmbE.

FIGURE S5. Identification of Glu-Ala and Ala-Glu-Ala as potential pathway intermediates bound to the T1 and T2 domains of AmbE.

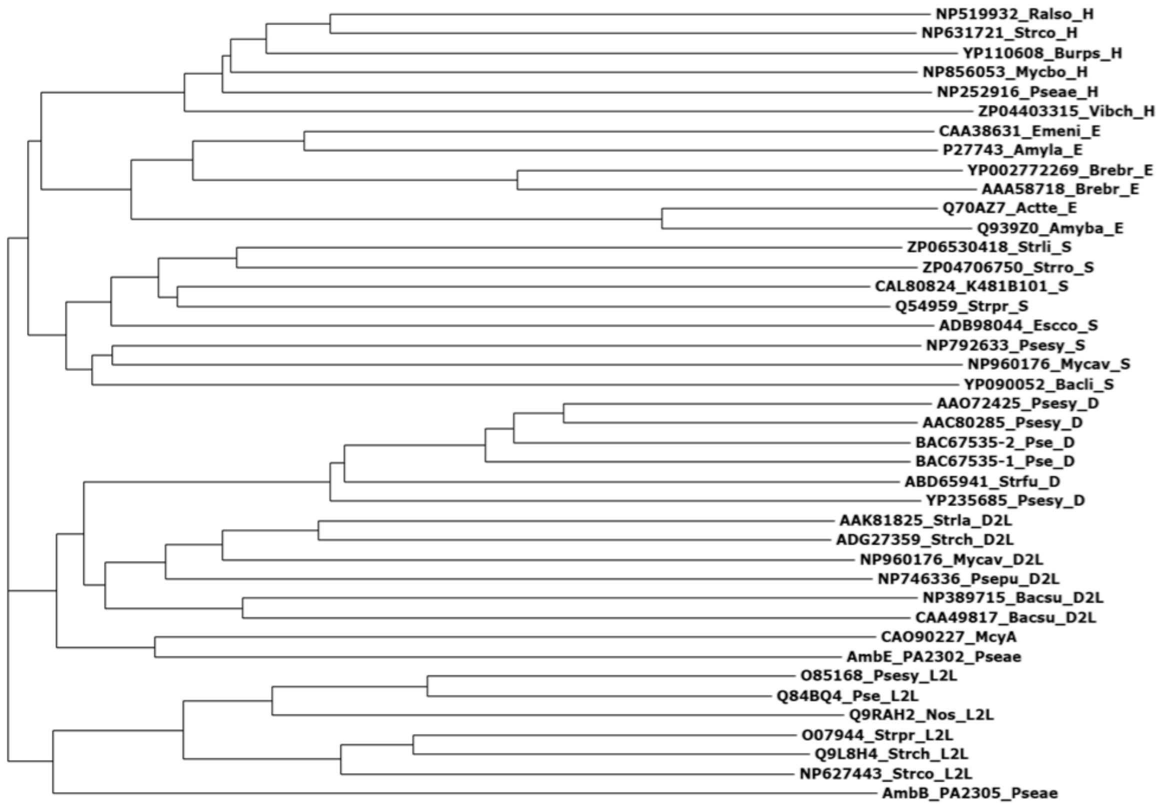

FIGURE S1. Phylogenetic analysis of the C* domain from AmbE. Condensation domain sequences were collected to be representative of the different condensation domain types. Sequences are identified by their accession number and annotated as follows: H = heterocyclization, E = epimerization, D = dual condensation/epimerization, L2L = condensation of L amino acids to L amino acids, and D2L = condensation of D amino acids to L amino acids, and S = starter). Sequences were compared using the Clustal Omega (Sievers et al., 2011; http://www.ebi.ac.uk/Tools/msa/clustalo/) with default parameters, and the subsequent Newick format of the neighbour-joining (NJ) tree without distance corrections was visualized using Newick Viewer (Boc et al., 2012; http://www.trex.uqam.ca/index.php?action=newick).

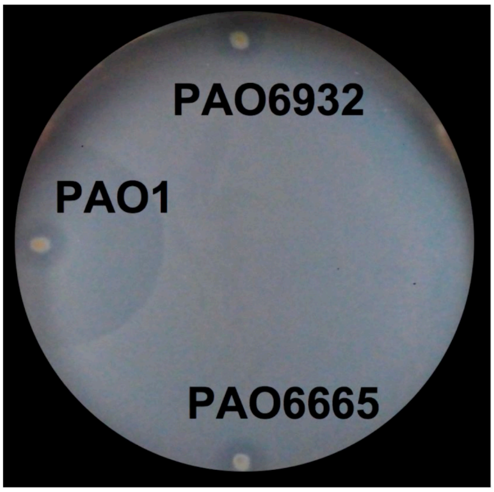

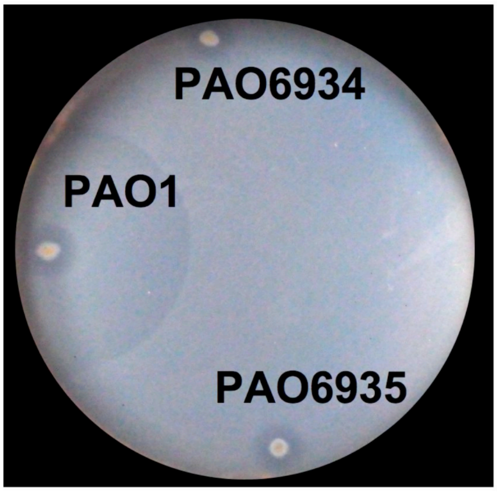

FIGURE S2. Biotest for AMB production by different *P. aeruginosa* strains. AMB is detected by a typical clearing zone around the *P. aeruginosa* colonies resulting from growth inhibition of the *E. coli* K-12 indicator strain. Strains used were PAO1 (wildtype), PAO6665 (AMB-negative control strain; Lee et al., 2010), PAO6932 (specifying $AmbB_{S768A)}$, PAO6934 (specifying $AmbE_{S1286A)}$, and PAO6935 (specifying $AmbE_{S1819A}$).

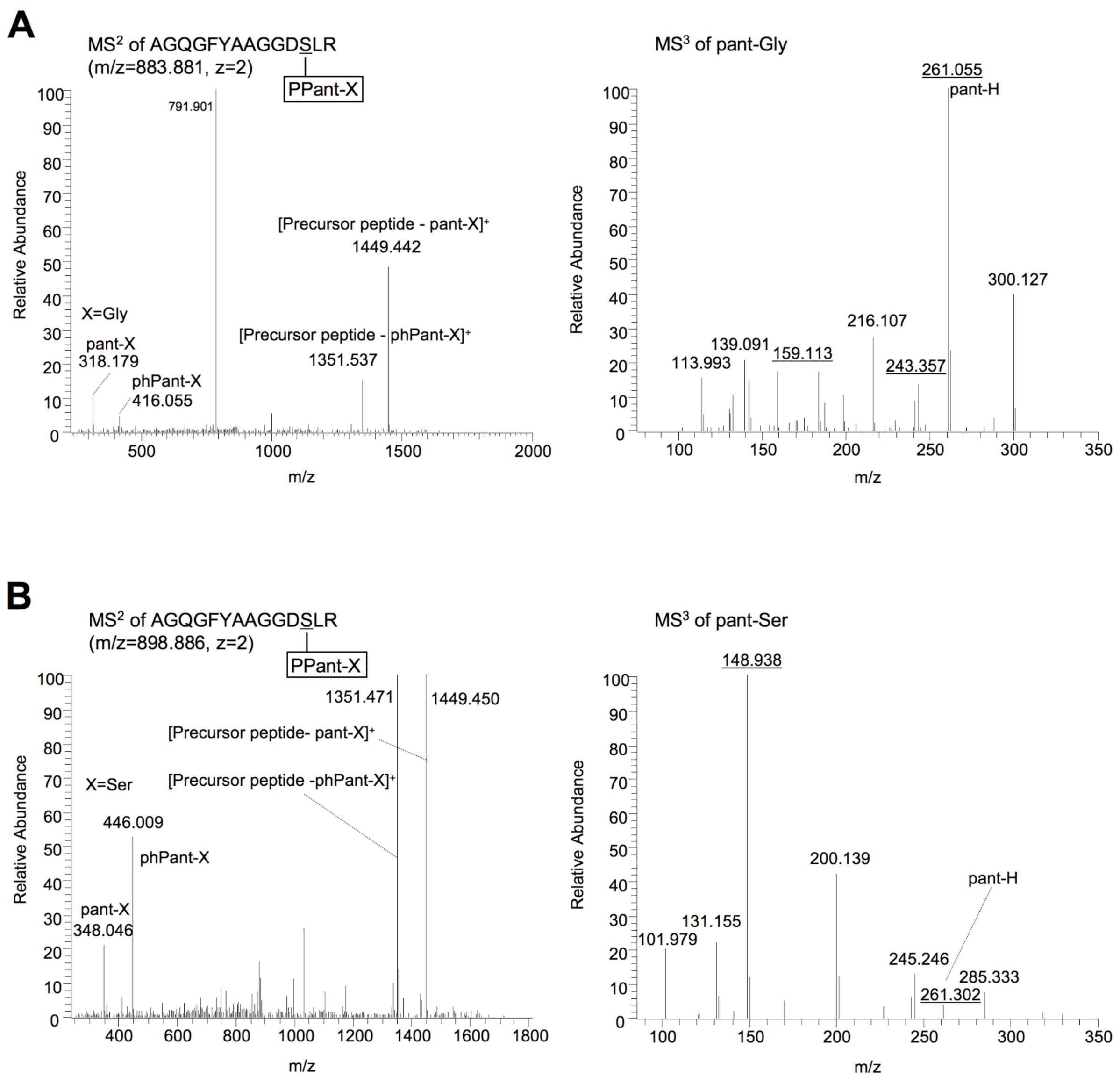

FIGURE S3. Identification of AmbB-bound Gly (A) and Ser (B) by phosphopantetheinyl elimination reactions. AmbB and AmbE (at 2.5 µM; converted to their holo-forms by Sfp) were incubated with 0.5 mM of all 20 proteinogenic amino acids before trypsin digestion and analysis by mass spectrometry.

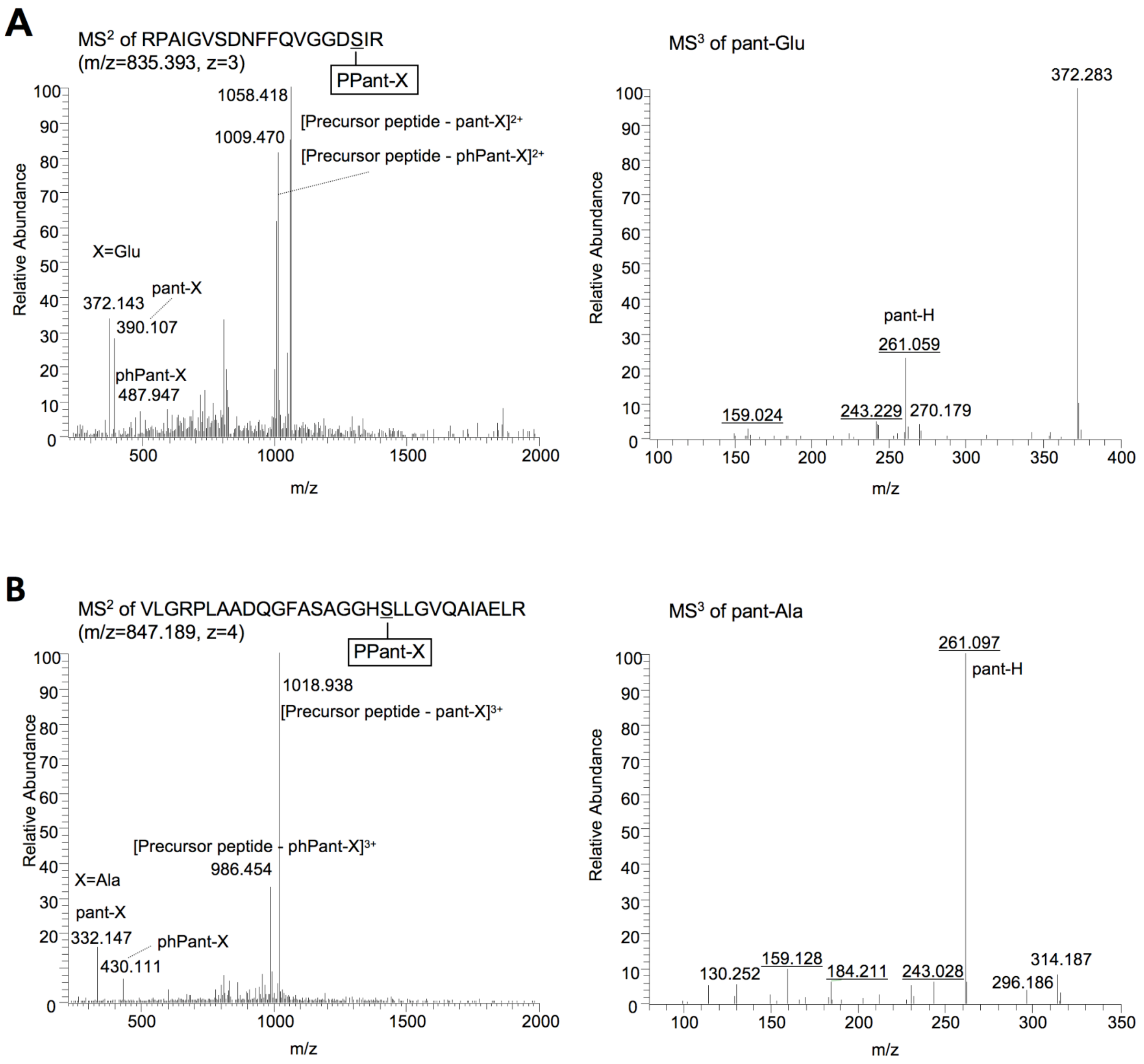

FIGURE S4. Identification of the amino acid substrates bound to the T1 and T2 domains of AmbE. AmbB and AmbE (at 2.5 µM; converted to their holo-forms by Sfp) were incubated with 0.5 mM of all 20 proteinogenic amino acids before trypsin digestion and analysis by mass spectrometry. Phosphopantetheinyl elimination reactions allowed identification of Glu bound at T1 (**A**) and of Ala bound at T2 (**B**).

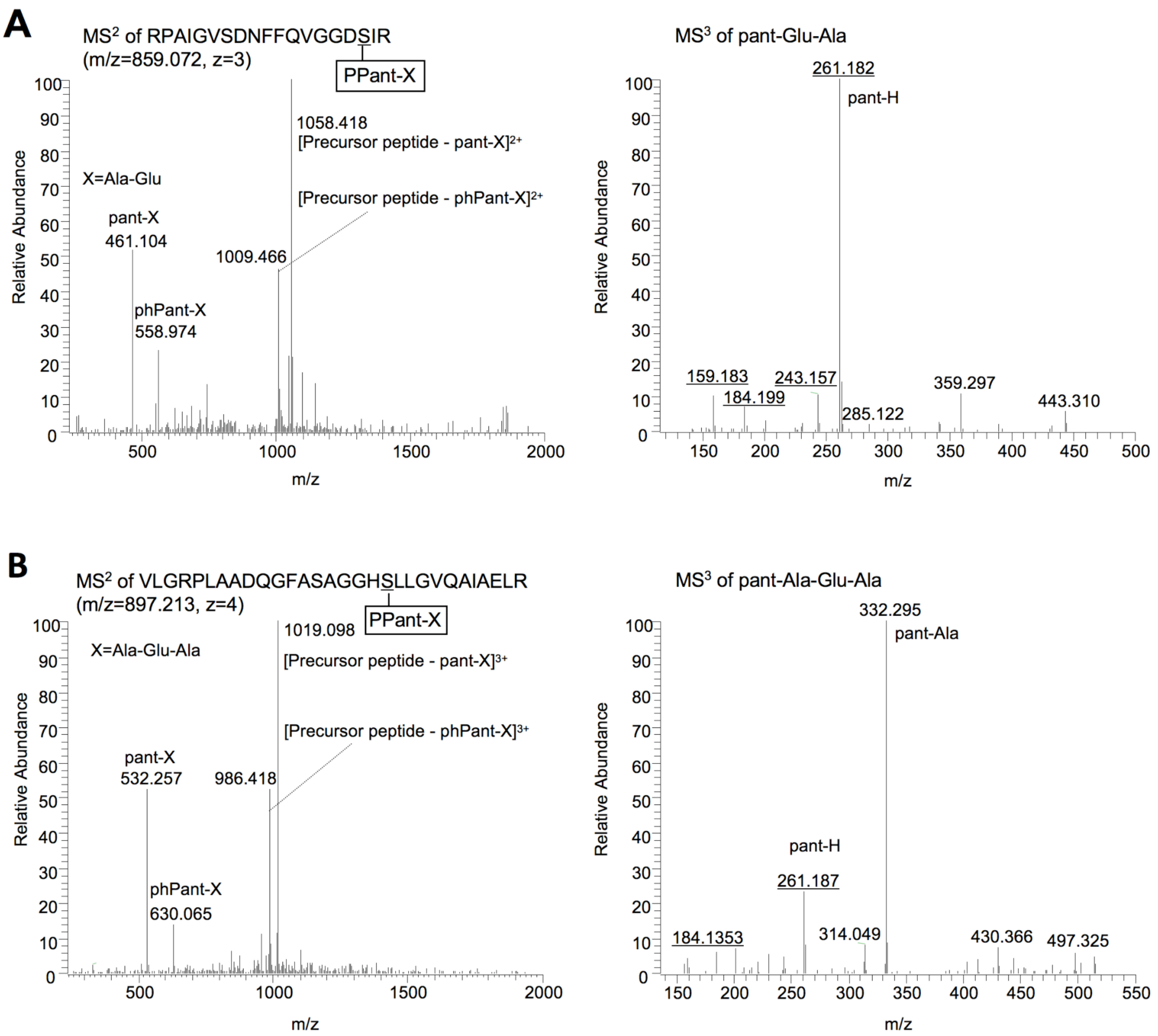

FIGURE S5. Identification of Glu-Ala and Ala-Glu-Ala as potential pathway intermediates bound to the T1 and T2 domains of AmbE. AmbB and AmbE$_{S1958A}$ (at 2.5 µM; converted to their holo-forms by Sfp) were incubated with L-Ala and L-Glu (at 1 mM) before trypsin digestion and analysis by mass spectrometry.
